# IgG4-related disease: a contemporary review

**DOI:** 10.3906/sag-2006-375

**Published:** 2020-11-03

**Authors:** Hazan KARADENİZ, Augusto VAGLIO

**Affiliations:** 1 Division of Rheumatology, Departmentof Internal Medicine, Faculty of Medicine, Gazi University, Ankara Turkey; 2 University of Florence and Meyer Children’s Hospital, Florence Italy

**Keywords:** IgG4-related disease, clinico-pathological characteristics, diagnosis, treatment

## Abstract

**Background/aim:**

Immunoglobulin G4-related disease (IgG4-RD), is an immune-mediated fibroinflammatory condition, which may involve multiple organs and mostly presents with high serum IgG4 levels and specific histopathological characteristics. As IgG4-RD is a relatively new entity the etiology, prevalence and epidemiologic knowledge is quite limited. Although involvement of almost all anatomical regions has been reported, the most commonly affected regions are pancreas, lacrimal glands, salivary glands, retroperitoneum, orbita, lymph nodes, kidney and lungs. Diagnosis is made with combined evaluation of clinical, radiological and histopathological findings. Typical histopathological features include storiform fibrosis, dense lymphoplasmacytic infiltrates and obliterative phlebitis. Its course is typically marked by remission and relapsing attacks and it may lead to fibrosis, destructive lesions in tissues and organ failure unless promptly treated. In the treatment of IgG4-RD, many approaches including surgical resection of tissues, systemic glucocorticoids, steroid-sparing immunosuppressive drugs, and biological agents are employed. Although association is not clear, malignancies are frequently reported in IgG4-RD patients. Therefore, it is prudent to monitor patients for the symptoms of malignant diseases.

**Conclusion:**

In this review, recent advances in clinico-pathological characteristics, diagnosis, and treatment of IgG4–RD are discussed.

## 1. Introduction

Immunoglobulin G4 related disease (IgG4-RD), is an immune-mediated fibroinflammatory condition, which may involve multiple organs and mostly presents with high serum IgG4 levels and specific histopathological characteristics. The concept of IgG4-RD was originally reported by Hamano et al. in 2001 with the rise in serum IgG4 levels in patients with autoimmune pancreatitis (AIP) [1]. Its etiology and triggering factors are still unclear. It occurs most commonly in middle aged and elderly males, but preponderance of male sex may vary according to the involve sites. Although involvement of almost all anatomical regions has been reported, the most commonly affected regions are pancreas, lacrimal glands, salivary glands, retroperitoneum, orbita, lymph nodes, kidney and lungs [2]. IgG4 **-** RD is often recognized incidentally during radiological or histopathological investigation of a tissue. Diagnosis is made with combined evaluation of clinical, radiological and histopathological findings. Its course is typically marked by remission and relapsing attacks and it may lead to fibrosis, destructive lesions in tissues and organ failure unless promptly treated. It also leads to pressure findings, secondary sclerosis and obstruction owing to tumefactive lesions [3,4]. In the treatment of IgG4-RD, many approaches including surgical resection of tissues, systemic glucocorticoids, steroid-sparing immunosuppressive drugs, and biological agents are employed. The spectrum of differential diagnoses includes malignancies, infections, autoimmune diseases, ANCA-associated vasculitis and Erdheim-Chester disease. In this review, recent advances in clinico-pathological characteristics, diagnosis, and treatment of IgG4–RD are discussed.

### 1.1. Epidemiology 

The precise prevalence of IgG4-RD is not known, due to being relatively a new entity and largely underrecognized in clinical practice. The disease occurs mostly in middle aged and elderly males, peaking between the 5th and the 7th decade. Studies have shown a male/female ratio varying between 1.6:1 to 4:1[4].A single study from Japan, reported a lower male/female ratio, i.e. 1:0.77 [5]. Type-1 AIP (IgG4-related), retroperitoneal fibrosis and IgG4-related tubulointerstitial nephritis occur more commonly in males, whereas head and neck involvement (e.g., thyroiditis, sialadenitis, and dacryoadenitis) is more common in females [6]. Certain studies revealed higher IgG4 serum levels and increased risk of recurrence in men [6]. In a study, it was observed that patients of Asian origin had higher IgG4 concentrations than patients of Western origin. The disease can also occur in pediatric age groups and shares characteristics with its adult **-** onset counterpart. In children, IgG4-RD presents with dacryoadenitis and mostly as an orbital mass [7].

### 1.2. Etiology 

The exact etiology of IgG4-RD still remains to be elucidated. However, some susceptibility genes and environmental factors have been described. Kawa et al. reported that HLA-DRB1*0405, HLA-DRB1*0401 haplotypes were associated with type-1 AIP in Japanese population [8]. As to Korean population, HLA-DQB1*0202, HLA-DRB1*0701 haplotypes were found to be associated with type 1 AIP [9]. In a large case **-** control study, retroperitoneal fibrosis was found to be associated with HLA **-** DRB1*03, a marker of many autoimmune disease [10].

In a few studies, autoantigens such as galectin-3, laminin 11, and annexin A11 have been reported, the presence of which, probably causes the triggering of condition **[** 11–13]. Estrogen is found as a risk factor in patients with IgG4-related mastitis. Due to eosinophilia in peripheral blood and involved tissues, an allergic etiology has also been considered [14]. Atopy/allergy occursin 20%–60% of IgG4-RD patients. A recent study form the Netherlands found increased occupational substance exposure among IgG4-RD during their lifetime including solvents and metal dusts [15]. Finally, an Italian case **-** control study showed asbestos exposure and smoking as risk factors for retroperitoneal fibrosis [16].

### 1.3. Histopathological characteristics 

**Figure 1:**
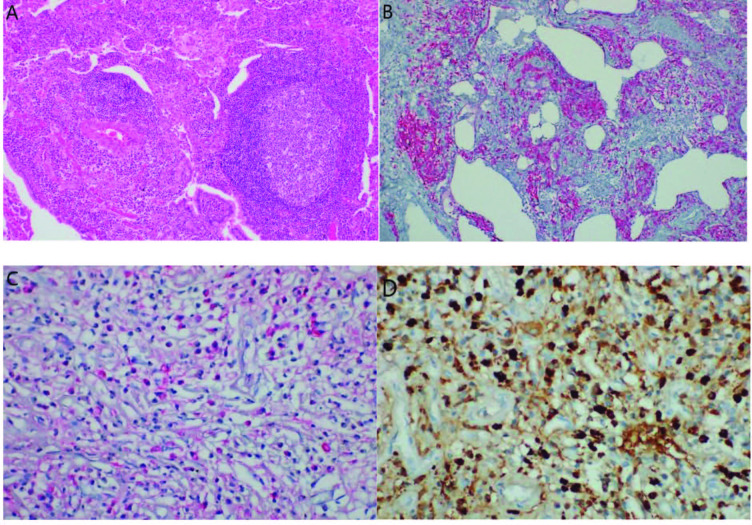
Characteristic histopathologic features of IgG4-RD. (A–D) IgG4-RD of the lung. (A) Lung parenchyma showing dense lymphoplasmocytic infiltration with lymphoidfollicles. (B) CD138 positive plasma cells. (C, D) IgG+ positive plasma cells. (Image courtesy of Nalan AKYÜREK).

Similar pathological features are observed across the affected tissues [17]. General pathological findings include dense lymphoplasmacytic infiltrates consisting of IgG4+ plasma cells, storiform fibrosis, obliterative phlebitis and mild to moderate eosinophilia [17]. The most specific findingis obliterative phlebitis, complete or partial obliteration of venous vessels, characterized by infiltration of vessel wall or lumen with dense lymphoplasmacytic infiltrate, composed of lymphocytes and plasma cells. Complete obliteration of the lumen can take place due to cell accumulation and fibrosis. After obliteration, venous channels are not visible with standard hematoxylin and eosin (H&E) stain, hence require specific elastin staining. Luminal fibrosis without inflammatory cells is not specific for IgG4-RD and it should suggest other diagnoses such as organized thrombus. Phlebitis not causing obliteration may also occur, but it is neither sensitive nor specific for IgG4-RD. Arterial vessels may also be affected in addition to venous vessels, but not as severely. Arterial involvement occurs mostly in cases with lung involvement and autoimmune pancreatitis but, it is rare in IgG4-RD and its presence does not rule out the diagnosis [18]. In IgG4-RD, necrosis, neutrophil **-** dominant infiltration and granuloma formation are absent, and if they are present, this is one of the exclusion criteria according to 2019 classification criteria [19]. If the above findings are present, a different accompanying disease such as vasculitis should be considered. Obliterative phlebitis or storiform fibrosis are rare in IgG4 associated lacrimal gland, salivary gland, lymph node and lung involvement, therefore they are not common features of IgG-RD. Another significant pathological finding is storiform fibrosis (an irregularly cartwheel like fibrotic pattern) **.** Collagen fibrils are interspersed within fibroblasts and myofibroblasts. Dense lymphoplasmacytic infiltration comprises mature, immature plasma cells and small lymphocytes (Figures 1A–1D). Lymphocytes are mostly CD4+T lymphocytes and spread throughout the lesion. B cells are found in scattered small lymphoid aggregates and ectopic germinal centers.

In addition, eosinophils are present at mild or moderate degree and rarely macrophages and histiocytes may be present. In conditions when eosinophils are preponderant, eosinophilic angiocentric fibrosis or eosinophilic cholangitis should be primarily considered. The degree of fibrosis compatible with IgG4-RD and IgG4 + plasma cell per high power field (HPF) cut-off number of IgG4 positive cells may vary from tissue to tissue. Although these numbers have not been verified yet, depending on the involved organ, cut-off value may range between 10–200 /HPF. In some organs such as the kidney, 10 IgG4+ / HPF and in salivary and lacrimal glands, >100 /HPF is considered as cut-off value. IgG4 /IgG+ plasma cell ratio provides stronger evidence for the diagnosis of IgG4-RD than IgG4 +plasma cell number. Cut off value for IgG4+/IgG+ plasma cell ratio is >40%. 

In many neoplasms, increasing number of IgG4+ plasma cells can be found both intratumorally and peritumorally. InIgG4-RD, plasma cells are polytypic. Typical electrophoretic pattern of a patient with IgG4-RD demonstrates polyclonal hypergammaglobulinemia with beta gamma bridging. Monotypic plasma cells are mostly seen inlymphoma and plasma cell malignancies [20]. A thorough physical examination may reveal the optimal sites for tissue sampling (i.e. major salivary gland swelling). Needle biopsies and fine-needle aspiration are usually inadequate for the diagnosis of IgG4-RD as they may not be able to detect storiform fibrosis and obliterative phlebitis. Yet, they may yield enough tissue to reliably rule out lymphoma and other malignant conditions. Nevertheless, for a firm diagnosis, pathological findings should always be supported by clinical, serological and/or radiological features.

### 1.4. Pathophysiology 

IgG4-RD is classified as a fibroinflammatory disorder, with involvement of both adaptive and innate immune mechanisms in its pathophysiology. IgG4 accounts for 1%–4% of IgG subtypes [11] and IgG4 antibodies are specific to mankind. IgG4 antibodies are considered to have antiinflammatory activity given the fact that they can undergo Fab-arm exchange and limit immune-complex formation. Due to the antiinflammatory properties of IgG4, IgG4 releasing plasmablasts represent a regulatory response to inflammatory stimuli. In spite of increased serum IgG4 level and tissue IgG4+plasma cells, which are characteristic of IgG4-RD, abnormal T cell activity is considered as the principal immune defect. Most lymphocytes present in the affected tissues of patients with IgG4-RD are T lymphocytes. Multiple studies have showed the involvement of follicular T helper (Tfh) lymphocytes and CD4+ cytotoxic T lymphocytes (CTL) in the pathogenesis of the disease [21]. It was observed that Tfh cells increase in affected tissues and peripheral blood of patients. In some studies, it was observed that IgG4-RD associated Tfh cells, induce differentiation and proliferation of B cells at a higher degree than normal Tfh cells in tissues [22]. Studies on subtypes of Tfh cells have indicated that,cells without CXCR3 and CCR6 expression, release Th2 type cytokines (IL-4,IL-5,IL-13) [23]. IL-4, a Tfh cytokine, induces germinal center development, B cell differentiation, plasmablast induction and class switch recombination, culminating in IgG4 production [24]. Many studies have demonstrated that progression of fibrosis is maintained via IL-4, IL-5, IL-13 cytokines [25]. IL-5 and IL-13 increase IgE and IgG4 production and lead to eosinophilic infiltration. In AIP, an increase in CD4+CD25+ Treg cells was found [26]. Treg cells contributeto IgG4 class switching and fibrosis respectively by releasing IL-10 and TGF-B [27]. Recent studies have stressed the importance of CD4+ CTL T lymphocytes in the physiopathology of the disease [28]. Matto et al. demonstrated oligoclonal expansion of CD4+ effector /memory CTL cells in both blood and tissues of IgG4-RD patients [29]. These CTL T cells contribute to the development of fibrosis in association with the mediators they release such as granzyme A, perforin, interleukin-1 β (IL-1 β), transforming growth factor -beta (TGF-β) and interferon-gamma (IFN-γ). Besides, cytotoxic T cells express F7 (SLAMF7) molecule on their surface, which contributes to fibrosis and is not present in other T cells. [28]. In patients with active IgG4-RD, rituximab leads to reduction in CD4+CTL cells secondary to B cell depletion [29]. In addition to T cells IgG4+ hypermutated plasmablasts have also been described in the blood of patients with active IgG4-RD [30].

## 2. IgG4-related disease and clinical findings

IgG4 **-** RD is usually a systemic disease, but a recent cluster analysis identified the following main phenotypes: pancreato-hepato-biliary disease, retroperitoneal fibrosis and/or aortitis, head and neck limited disease, classical Mikulicz syndrome with systemic involvement [31]. The main organ manifestations are discussed below.

### 2.1. IgG4-related lymphadenopathy 

Although lymphadenopathy usually occurs in conjunction with other clinical and laboratory findings of the disease, sometimes it may be the initial or sole finding. There are 5 histological patterns and nodal inflammatory pseudotumor-like represents the most specific pattern for IgG4-RD (Table 1). Lymph nodes are usually, nontender and have elastic consistency. Symptoms usually emerge due to mass effect by enlarged lymph nodes. Usually, more than one lymph node group is affected. Mediastinal, hilar, intraabdominal and axillary lymph nodes are the most commonly involved. As storiform fibrosis, one of the characteristic features of IgG4-RD, occurs rarely and IgG4 plasma cells stain positive in many diseases, making diagnosis solely with lymph node biopsy is quite challenging. While a true lymphadenitis is characterized by the presence of IgG4+ plasma cells in interfollicular regions, their presence in intrafollicular regions is nonspecific. The differential diagnosis of generalized lymphadenopathy includes sarcoidosis, multicentric Castleman disease, infections (e.g., tuberculosis, HIV infection), lymphoma and other malignant conditions. IgG4 related lymphadenopathy is distinguished from other lymphadenopathies by its mild lymph node enlargement, typical characteristics in biopsy, absence of constitutional symptoms, and response to glucocorticoids [32].

**Table 1:** IgG4-related lymphadenopathy histological patterns.

(i) Multicentric Castleman disease-like
(ii) Follicular hyperplasia
(iii) Interfollicular expansion
(iv) Progressive transformation of germinal center-like
(v) Nodal inflammatory pseudotumor-like

### 2.2. IgG4-related pancreatitis

There are two types of autoimmune pancreatitis (AIP). The IgG4-RD associated AIP is called as type 1 AIP, while the IgG4 **-** unrelated AIP is type 2 AIP or idiopathic duct-centric chronic pancreatitis (IDCP) or granulocyte-positive epithelial pancreatitis. Type 2 AIP occurs much more rarely than type 1 AIP and is sometimes associated with inflammatory bowel disease. Types 1 and 2 AIP display different clinical features. Type 1 AIP occurs mostly in elderly males and is less likely to cause jaundice and abdominal pain. Type 2 AIP affects mostly younger people without any sex predilection. In addition, its rate of recurrence is lower and is confined to the pancreas. In the majority of type 1 AIP patients, extrapancreatic IgG4-RD involvement occurs. Development of endocrine and exocrine pancreatic insufficiency manifesting with hyperglycemia and steatorrhea is not uncommon among patients who suffer from wither type of AIPs. In some patients with type 1 AIP, acute recurrent chronic pancreatitis may occur. Type 1 AIP may imitate pancreatic carcinoma as it leads to mass **-** like lesions of the pancreas. It is also difficult to clinically discriminate AIP patients from those with adenocarcinoma since painless obstructive jaundice and high serum IgG4 concentrations may be seen in both conditions [33]. Many AIP patients had to undergo radical surgery due to concern about pancreatic cancer. In CT images of type 1 AIP patients, enlarged pancreas, sausage **-** like pancreas, loss of lobulation, irregular pancreatic duct, hypodense rim/halo sign (edema surrounding the organ in the form of halo) and minimal peripancreatic fat stranding are observed. Magnetic resonance cholangiopancreatography (MRCP) images of patients with IgG4 autoimmune pancreatitis show nonvisualization of a long segment of the main pancreatic duct. Patients who are left untreated or have long term AIP can develop symptoms of chronic pancreatitis with atrophic pancreas and intraductal calcifications on imaging [34].

### 2.3. IgG4-related sclerosing cholangitis (IgG4-RSC)

IgG4 **-** RSC usually produces obstructive jaundice, weight loss and/or steatorrhea and elevation in hepatic function tests [35]. IgG4-RSC is different from primary sclerosing cholangitis (PSC) which occurs at younger ages in association with inflammatory bowel disease and histopathologic examination shows circumferential or onion-skin like fibrosis and in advanced stages, ductopenia [36]. Unlike PSC, IgG4-RSC tissue biopsy yields IgG4 + plasma cell infiltrates, severe interstitial fibrosis, transmural thickening and more severe portal and lobular inflammation [36]. Other differences are increased serum IgG4 concentration and characteristic response to corticosteroids. IgG4-RSC is the most common extra pancreatic disease in patients with type 1AIP, with a prevalence of 70%. It seldomly appears in the absence of pancreatitis. Considering the difficulties in making the diagnosis of isolated IgG4-RSC in the biliary system, it is not easy to estimate its actual prevalence and to appreciate its severity [37]. IgG4-RSC can also be confused with cholangiocarcinoma, but the latter has higher serum bilirubin and CA19-9 levels [38]. CT findings of IgG4-RSC include multiple focused strictures, >2.5 mm thickening in bladder wall, and long segment narrowing. These strictures are smooth in morphology, with resultant proximal biliary tree dilatation. Posttreatment imaging may reveal radiologic improvements in strictures and biliary tree wall thickening [39].

### 2.4. IgG4-related salivary gland involvement 

The involvement of major salivary (parotid and submandibular) glands is a common feature of IgG4-RD. Lacrimal or parotid gland enlargements, which was previously termed as Mikulicz syndrome, and Küttner tumor (chronic sclerosing sialadenitis) have been incorporated into IgG4-RD context [40]. In IgG4-RD, lacrimal gland, parotid gland and submandibular gland are usually involved together at different combinations. Although it is not always symmetrical, glands are usually involved bilaterally. Renal involvement with low complement levels might be encountered in this condition [4].

In the morphology of IgG4-related sialadenitis, fibroinflammatory infiltration occurs in interlobular septa, but lobular architecture is preserved, and commonly hyperplastic irregular lymphoid follicles are detected [41]. In some sialadenitis cases due to stones, IgG4 positive cells may be present. In a study, serum IgG4 levels were found elevated in 7.5% of patients with primary Sjögren syndrome (pSS). Compared to pSS, in IgG4-RD have higher serum IgG4 levels and negative antinuclear, anti-Ro/SSA and anti-La/SSB antibodies [42]. Radiologically, pSS demonstrates the “apple-tree sign” on sialography, indicative of contrast spilling from degenerated glands. Both pSS and IgG4-RD enlarge lacrimal and salivary glands, but dryness in mouth and eyes is milder in IgG4-RD and more severe in pSS due to more extensive epithelial cell apoptosis. Upon immunosuppressive treatment IgG4-RD patients regain their salivary functions whereas, pSS often leads to irreversible destruction of salivary units.

### 2.5. IgG4-related dacryoadenitis and orbital inflammatory disease 

IgG4-RD presenting with the involvement of lacrimal glands (IgG4-related dacryoadenitis) [43] and orbital involvement occurs at the rate of 17%–23% [44]. IgG4 related orbital inflammatory disease commonly involves lacrimal glands, while involvement of orbital soft tissue (IgG4-related sclerosing orbital inflammation), extraocular muscles, palpebrae, optical nerve, or orbital bone have also been reported. Approximately 70%–80% of patients have extra ophthalmic findings. Some studies have suggested that it is more common in females [45], whereas some other studies reported that it is more common in males [46]. Patients may present with symptoms of exophthalmos, periocular swelling and mass which can lead to proptosis and compression of local nerves. IgG4 related dacryoadenitis may lead to blindness unless treatment is rapidly commenced [47]. Fibrosis occurs mostly in interlobular septa and causes impairment of the architecture of lacrimal gland [48]. The differential diagnosis of IgG4 related orbital disease includes Sjögren’s syndrome, granulomatosis with polyangiitis, sarcoidosis, lymphoma, infection, orbital pseudotumor and idiopathic orbital inflammation [48] (Table 2). IgG4-RD accounts for 25%–50% of orbital pseudotumor. In addition, depending on diagnostic criteria used, it also accounts for 5%–25% of idiopathic orbital inflammations [49]. In a study, 38 patients diagnosed with chronic dacryoadenitis or orbital pseudotumor, 15(39%) met the definite criteria of IgG4-RD, while 5 patients had probable disease [50]. Some other studies have demonstrated that IgG4-RD itself, produces susceptibility to ocular adnexal mucosa associated lymphoid tissue (MALT) lymphoma and other lymphomas [51].

**Table 2:** Differential diagnosis of IgG4-related disease.

***Autoimmune disorders*** ***-Rheumatic conditions*** Sarcoidosis Sjogren’s syndrome ANCA-associated vasculitis -Granulomatosis with polyangiitis -Eosinophilic granulomatosis with polyangiitis -Microscopic polyangiitis Takayasu’s arteritis-giant cell arteritis Behçet disease ***-Nonrheumatic conditions*** Primary biliary cirrhosis-primary sclerosing cholangitis Autoimmune hepatitis Hashimoto’s thyroiditis Castleman’s disease Lymhomatoid granulomatosis	Infections Bacterial Viral Mycobacterial Spirochetal Fungi ***Lymphoproliferative disorders*** MALT lymphoma with plasmocytic differentiation Plasma cell neoplasia Follicular lymphomas
***Systemic inflammatory disorders*** Rosai-Dorfman disease Multicentric Castleman disease Chronic sialadenitis Type 2 AIP Inflammatory bowel disease Erdheim-Chester disease	***Tumors*** Inflammatory myofibroblastic tumor Adenocarcinoma and squamous cell carcinoma ***Eosinophilic disorders*** Eosinophilic angiocentric fibrosis Kimura disease Anjiolymphoid hyperplasia with eosinophilia

### 2.6. Retroperitoneal fibrosis and related diseases 

**Figure 2:**
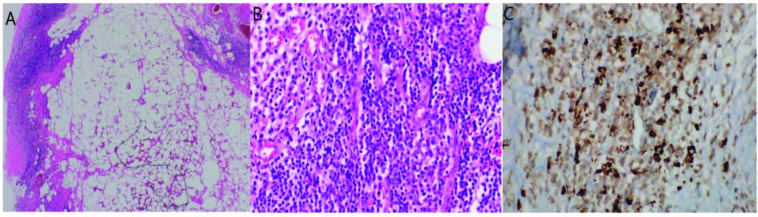
Characteristic histopathologic features of IgG4-RD. (A–C) IgG4-RD of the omentum. (A) Omental biopsy showing fat necrosis areas and peripherally located dense lymphoplasmocytic infiltrate. (B) Higher power view of dense lymphoplasmocytic infiltrate. (C) IgG4 positive plasma cells in one high power field. (Image courtesy of Nalan AKYÜREK).

Retroperitoneal fibrosis (RPF) is one of the most commonly encountered subtypes of IgG4-RD. RPF may be idiopathic (Ormond disease) or may have a secondary cause (Table 3). Idiopathic forms account for 70% of cases and may be of IgG4-RD or non-IgG4-RD origin. In a study, it was established that IgG4-RD was responsible for many of RPF cases, which were previously considered idiopathic [52]. Asbestosis and tobacco exposure increase RPF risk [16]. Patients usually present with urinary obstruction and kidney failure and frequently complain of lower back, flank and abdominal pain due to urinary obstruction [53]. In two large retrospective studies, pain was evident in more than 90% of patients on presentation [54,55]. Pain is dull and not well localized and does not change with activity or body position. Sometimes, the pain may be acute/sharp resembling that of a renal colic. In a study performed in North America, many cases of retroperitoneal fibrosis were identified during radiologic examinations performed for the suspicion of nephrolithiasis [56]. Due to inflammatory nature of lesion, pain responds better to NSAIDS than opioids. In a study conducted on 40 patients, testicular pain was reported in more than 50% of male patients [57]. Kidney atrophy may be seen as a result of unilateral chronic obstructive uropathy or renal artery stenosis caused by RPF. Patients may exhibit edema, thrombophlebitis or deep vein thrombosis in lower limbs due to obstruction of inferior vena cava. In a study conducted in Netherlands, hydrocele was detected in 29% of patients [55]. Serum creatinine and urea levels may rise secondary to urinary obstruction. High acute phase reactants are usually associated with a more symptomatic disease however they respond poorly to treatment and usually do not increase during flares. The initial imaging modality employed for investigating urinary pathologies is ultrasonography [58]. For visualization of pancreas and retroperitoneal organs CT or MRI is preferred. Histological confirmation of RPF may be required if the mass lesion has atypical localization, and clinical and laboratory findings suggestive of infection or malignancies. During surgical exploration, macroscopically, a hard, white plaque of varying thickness is seen. Microscopically, fibrous tissue contains extracellular matrix which is organized in thick irregular bundles composed of type 1 collagen fibrils surrounding small retroperitoneal vessels [59] (Figures 2A–2C).

**Table 3:** Main causes o secondary retroperitoneal fibrosis.

Drugs	Malignancy	Infections
Ergot derivatives	· Carcinoid tumor	· Tuberculosis
· beta blockers,	· Hodgkin’s lymphoma	· Histoplasmosis
· bromocriptine,	· Non-Hodgkin’s lymphoma	· Actinomycosis
· hydralazine,	· Retroperitoneal sarcoma	
· methysergide,	· Breast cancer	Histiocytosis (Erdheim-Chester disease)
· Biologicals	· Colorectum cancer	Secondary (AA) amyloidosis
-etanercept	· Prostate cancer	Radiation therapy
-infliximab	· Bladder cancer	Surgery/trauma

**Figure 3:**
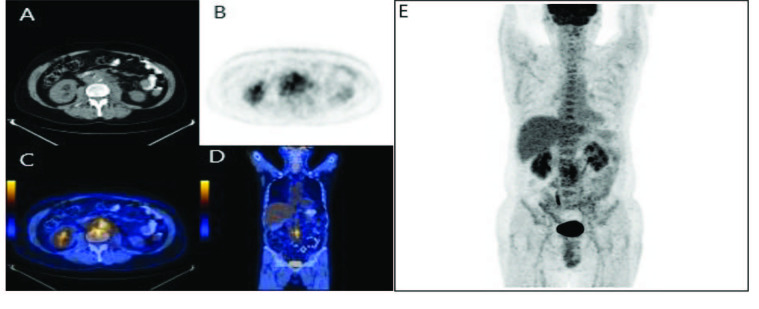
PET findings in RPF. 18F-FDG PET scans in a patient with idiopathic RPF show intense accumulation of ^18^F-FDG arround the abdominal aorta [A, axial CT, B, axial PET, C, axial fusion images, D, coronal fusion images, E, coronal maximum intensity projection (MİP) PET images)]. (Image courtesy of Özlem ATAY).

Typical CT findings, include medially displacement of ureter due to compressive effects of fibroinflammatory mass lesion involving surroundings of aorta and inferior vena cava18-FDG positron emission Tomography (18-FDG PET) is a beneficial and reliable technique for evaluating metabolic or inflammatory activity of disease. Serious complications such as renal failure may occur unless patients are treated promptly [60] (Figures 3A–3E).

The aim of treatment is to relieve obstruction caused by fibrosis, to halt progression of the fibrotic process and to prevent disease recurrence. If there is an important urinary tract obstruction, this should be eliminated with open surgery, percutaneous intervention or endoureteral approach. As soon as diagnosis is made (the same day as urological intervention) steroid treatment should be commenced. If there is no adequate response to steroids following approximately four weeks of treatment, immunosuppressive treatments should be instituted such as mycophenolate mofetil (MMF), azathioprine (AZA), methotrexate, cyclophosphamide, and cyclosporine [61]. Patients who have positive PET findings at the onset of treatment respond better to steroids than those who have negative PET findings [62]. If clinical or radiological improvement is not observed in spite of four weeks of medical treatment, repeat CT investigation and biopsy are recommended in order to corroborate the diagnosis.

### 2.7. IgG4-related renal involvement 

IgG4 **-** related renal involvement, most commonly occurs as tubulointerstitial nephritis (TIN). IgG4-related TIN may be detected during investigations carried out for the suspicion of kidney masses, abnormal urinary analysis, and/or renal failure. Histological findings include lymphoplasmacytic infiltration of the renal interstitium, tubular atrophy and fibrosis. In patients with IgG4-related TIN, low C3 and C4 levels, can be seen especially in patients with active disease. The cause of hypocomplementemia occurring in IgG4-TIN is not entirely clear, since IgG4 itself binds weakly to complement. Hence, it is thought that IgG1 and IgG3 account for hypocomplementemia. CT imaging shows enlarged kidneys and hypodense lesions. Apart from IgG4-TIN, membranous nephropathy and mesengioproliferative glomerulonephritis may also occur, albeit rare in IgG4-related kidney disease [63]. Membranous nephropathy can be concurrent with TIN and presents with nephrotic range proteinuria and hypoalbuminemia [63].

### 2.8. IgG4-related respiratory disease (IgG4-RRD)

IgG4-RRD may be detected incidentally with symptoms such as cough, dyspnea, and chest pain. Apart from dense lymphoplasmacytic infiltrates, small aggregates of neutrophils may be present in alveolar spaces or within the inflammatory infiltrates.The fibroinflammatory infiltrate typically extends towards perilymphatic region-bronchovascular tree and interlobular septa. 

Lung manifestations predominantly present as inflammatory pseudotumor, interstitial pneumonitis, organizing pneumonia, and lymphomatoid granulomatosis. IgG4-RD involves not only lung parenchyma, but also pleura, airways, mediastinum, and vasculature. There are four patterns of lung involvement [64] (Table 4).

**Table 4:** IgG4-related respiratory disease histological patterns.

(i) Solid nodular pattern Obliterative arteritis is often seen
(ii) Broncho vascular pattern Thickening of interlobular septa and bronchovascular bundles
(iii) Alveolar interstitial pattern Honeycombing, bronchiectasis, and widespread ground glass
(iv) Round-shaped ground glass opacity

Delayed diagnosis and treatment of IgG4-related interstitial pneumonia can lead to significant pulmonary fibrosis. Many patients exhibit more than one pattern. Visceral and parietal pleura thickening may also take place. In a recently published case-based review, IgG4-related pleural effusion has been reported in 17 patients and in pleural effusion sampling, adenosine deaminase levels were found to be high [65]. The differential diagnosis of IgG4-RRD include MCD, sarcoidosis, lymphoma and inflammatory myrofibroblastic tumor. The solid nodular type may be confused with sarcoidosis. Hilar lymphadenopathy is present in both sarcoidosis and IgG4-RD and they might be discriminated by PET CT, since CT alone is inadequate in this respect. In sarcoidosis fluorodeoxyglucose SUV max uptake on PET is higher. Ga-67 scintigraphy is inadequate in staging of the disease and evaluation of activity and response to treatment in comparison to PET CT [66].

In IgG4-RRD, the relation with malignancy depends on the pattern displayed, i.e. while the risk of malignancy is higher in those with interstitial and nodular patterns, it is not seen in those with bronchovascular pattern. In addition, prognosis is poorer in interstitial pattern. 

### 2.9. IgG4-related aortitis/periaortitis

**Figure 4:**
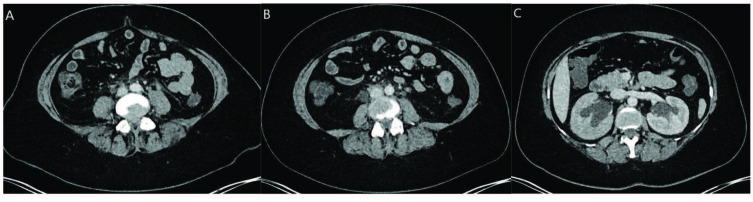
A and B, retroperitoneal fibrosis. Contrast-enhanced axial images (A) and coronal image (B) of computed tomography of the abdomen show heterogenous soft tissue densities surrounding aorta in the retroperitoneum at the infrarenal level. Soft tissue densities severely surround aorta, aortic bifurcation and main iliac arteries bilaterally (black arrow). (Image courtesy of Emetullah CİNDİL).

IgG4-RD is one of the causes of noninfective aortitis [67]. In retrospective pathological studies of patients undergoing aorta resection, a number of patients with thoracic lymphoplasmacytic aortitis, abdominal periaortitis and inflammatory abdominal aorta aneurysm were identified [68] (Figures 4A and 4B).Thoracic aortitis can lead to aneurysm formation or dissection, though it is often asymptomatic and detected incidentally by imaging. In a recent prospective study, in 89 out of 587 IgG4-RD patients (15.2%), periaortitis/periarteritis was detected, which was most commonly distributed in infrarenal abdominal aorta and iliac arteries. (83.5%) [69]. IgG4-RD may also involve medium sized vessels such as coronary arteries in addition to large vessels. CT angiography is observed the soft-tissue masses around the proximal segments of the left anterior descending (LAD) and right coronary artery (RCA). The presence “mistletoe sign” in CT angiography is an indicator of IgG4-RD associated coronary artery disease [70]. Mistletoe is a plant attached to the branches of a tree, similar to the perivascular soft, tissue attached to the coronary tree.

### 2.10. IgG4-related thyroid disease 

Riedel thyroiditis, which is a rare form of thyroiditis, is the IgG4-RD of thyroid gland. Stiff thyroid leads to dyspnea, dysphagia and hoarseness. Cytologic examination is not always diagnostic and diagnosis is usually made with thyroid resection, carried out to relieve clinical symptoms and to rule out malignancy. The fibrosing variant of Hashimoto thyroiditis has also been associated with IgG4-RD [71]. In comparison to IgG4-negative thyroiditis, IgG4-RD thyroiditis presents serologically with higher levels of antithyroglobulin and antithyroid peroxidase antibodies.

### 2.11. Other IgG4-related organ involvements

**Skin disease** (cutaneous pseudolymphoma, hyperplasia with eosinophilia, angiolymphoid hyperplasia with eosinophilia (ALHE)) **:**

IgG4 **-** RD should be considered as part of the differential diagnosis for nodules, papules, and plaques. Cutaneous lesions are predominantly located on the head and neck in IgG4 **-** RD cases (73.1%) [72]. IgG4 **-** related autoimmune hepatitis and hepatic inflammatory pseudotumor, constrictive pericarditis, sclerosing mastitis, gastritis are included among the rare IgG4 **-** RD manifestations.

## 3. Diagnosis

The diagnosis of IgG4-RD is currently based on the comprehensive classification criteria proposed in 2019 by Wallace et al. [19] (Table 5).

**Table 5:** The 2019 American College of Rheumatology/European League Against Rheumatism classification criteria for IgG4- RD [Wallace ZS et al. (2019)].

Step 1: Entry criteria (Yes† or No)	Step 2: Exclusion criteria:domains and items ‡ (Yes or No δ)
Characteristic*clinical or radiological involvement of a typical organ (e.g., pancreas, salivary glands, bile ducts, orbits, kidney, lung, aorta, retroperitoneum) orpathological evidence of an inflammatory process accompanied by a lymphoplasma cyticin filtrate of uncertain aetiology in one of these same organs.	Clinical · Fever · No objective response to glucocorticoids Serological · Leucopenia and thrombocytopenia with no explanation · Peripheral eosinophilia · Positive antineutrophilcyto plasmic antibody (specifically aganist proteinase 3 or myeloperoxidase) · Positive SSA/Ro or SSB/La antibody · Positive double-stranded DNA, RNP or Sm antibody · Other disease-specific autoantibody · Cryoglobulinemia Radiological · Known radiological findings suspicious for malignancy or infection that have not been sufficiently investigated Rapid radiological progression Long bone abnormalities consistent with Erdheim-Chester disease Splenomegaly Pathological · Cellular infiltrates suggesting malignancy that have not been sufficiently evaluated · Markers consistent with inflammatory myofibroblastic tumour · Prominent neutrophilic inflammation · Necrotizing vasculitis · Prominent necrosis · Primarily granulomatous inflammation · Pathologic features of macrophage/histiocytic disorder · Known diagnosis of the following: Multicentric Castleman’s disease Chrohn’s disease or ulcerative colitis (if only pancreatobiliary disease is present) Hashimoto thyroiditis (if only the thyroid is affected
If case meets entry criteria and does not meet any exclusion criteria proceed to Step 3 ¶	Step 4: Total inclusion points
Step 3. Inclusion criteria: domains and items Numerical weight Serum IgG4 concentration · Normal or not checked 0 · >Normal but <2x upper limit of normal +4 · 2-5x upper limit of normal +6 · ≥5x upper limit of normal +11 Bilateral lacrimal, parotid, sublingual and submandibular glands · Not set of glands involved 0 · One set of glands involved +6 · Two or more sets of glands involved +14 Chest · Not checked or neither of the items listed is present 0 · Peribronchovascular and septal thickening +4 · Paravertebral band-like soft tissue in the thorax +10 Pancreas and biliary tree · Not checked or neither of the items listed is present 0 · Diffuse pancreas englargement (loss of lobulations) +8 · Diffuse pancreas englargement and capsule-like rim +11 with decreased enhancement · Pancreas (either of above) and biliary tree involvement +19 Kidney · Not checked or neither of the items listed is present 0 · Hypocomplementemia +6 · Renal pelvis thickening/soft tissue +8 · Bilateral renal cortex low-density areas +10 Retroperitoneum · Not checked or neither of the items listed is present 0 · Diffuse thickening of the abdominal aortic wall +4 · Circumferential or anterolateral soft tissue around +8 the infrarenal aorta or iliac arteries Histopathology · Uninformative biopsy 0 · Dense lymphocytic infiltrate +4 · Dense lymphocytic infiltrate and obliterative phlebitis +6 · Dense lymphocytic infiltrate and storiform fibrosis +13 with or without obliterative phlebitis Immunostaining ** 0–16, as follows: · Assigned weight is 0 if: the IgG4+/IgG+ratio is 0%–40% or indeterminate and the number of IgG4+cells/hpf is 0–9 *** · Assigned weight is 7 if: (1) the IgG4+/IgG+ ratio is ≥41% and the number of IgG4+cells/hpf is 0–9 or indeterminate or (2) the IgG4+/IgG+ ratio is 0%–40% or indeterminate and the number of IgG4+cells/hpf is ≥10 or indeterminate. · Assigned weight is 14 if: (1) the IgG4+/IgG+ ratio is 41%–70% and the number of IgG4+cells/hpf is ≥10 or (2) the IgG4+/IgG+ ratio is ≥71% and the number of IgG4+ cells/hpf is 10–50.Assigned weight is 16 if: the IgG4+/IgG+ratio is ≥71% and the number of IgG4+cells/hpf is ≥51.	A case meets the classification criteria for IgG4-RD if the entry criteria are met, no exclusion criteria are present, and the total points is ≥20.

*Refers to enlargement or tumour-like mass in an affected organ except in (1) the bile ducts, where narrowing tends to occur, (2) the aorta, where wall thickening or aneurysmal dilatation is typical and (3) the lungs, where thickening of the bronchovascular bundles is common. †If entry criteria are not fulfilled, the patient cannot be further considered for classification as having IgG4-RD. ‡Assessment for the presence of exclusion criteria should be individualised depending on a patient’s clinical scenario. δ If exclusion criteria are met the patient cannot be further considered for classification as having IgG4-RD. ¶ Only the highest weighted item in each domain is scored. **Biopsies from lymph nodes, mucosal surfaces of the gastointestinal tract and skin are not acceptable for use in weighting the immunostaining domain. *** ‘Indeterminate’ refers to a situation in which the pathologist is unable to clearly quantify the number of positively staining cells within an infiltrate, yet can still ascertain that the number of cells is at least 10/hpf. For a number of reasons, most often pertaining to the quality of the immunostain pathologist Are sometimes unable to count the number of IgG4+ plasma cells with precision yet even so, can be confident in grouping cases into the appropriate immunostaining result category. hpf: high power field, IgG4-RD: IgG4-related disease.

Comprehensive classification criteria issued by 2019 ACR/EULAR involves three steps; in the first step, in a potential case, at least one of the 11 organs consistent with the definition of IgG4-RD should be demonstrated. In the second step, the exclusion criteria step, a total of 32 clinical, serological, radiological and pathological items should be considered. The presence of one of these criteria is enough to rule out IgG4-RD. In third step, 8 important criteria are considered, and serological results, radiological evaluations and pathological interpretations are addressed, scored and interpreted. Overall a score of 20 or more is diagnostic of IgG4-RD. Although comprehensive diagnostic criteria are commonly used, as it is difficult to take biopsy from regions such as pancreas, retroperitoneum and orbita, they can be used with difficulty in diseases of these regions. Thus, organ specific diagnostic criteria have been developed and can be employed instead of comprehensive diagnostic criteria [73,74].

### 3.1. Laboratory parameters

In many patients, serum IgG4 levels are high. Its level usually correlates with the severity of the disease. However, serum and tissue IgG4 concentrations are neither specific nor sensitive indicator of IgG4-RD. A study demonstrated that almost half of active patients with histologically proven IgG4-RD had normal serum IgG4 levels [4]. Involvement of certain organs or anatomic regions, particularly the retroperitoneum, have lower rates of correlation with serum IgG4 levels. False-negative IgG4 levels due to the prozone phenomenon should be considered in cases with multiorgan involvement and low serum IgG4. In addition, peripheral eosinophilia, high serum IgE levels, polyclonal hypergammaglobulinemia, high CRP, low titer positive antinuclear antibody, rheumatoid factor and hypocomplementemia are common in IgG4-RD. The serum IgG4 level decreases promptly after treatment with glucocorticoids or B cell depletion in most patients despite many patients do not achieve normal levels while they are in clinical remission. In a prospective trial of rituximab in IgG4-RD, higher baseline levels in serum IgG4, IgE and blood eosinophil concentrations predicted greater risk of IgG4-RD relapse and shorter time to relapse, rendering monitoring of these values significant [75]. Apart from IgG4,in a recent study, in the sera of 28 IgG4-RD patients, thymus and activation-regulated chemokine (TARC) levels were measured and were found to be higher than those of patients with Sjögren syndrome and control group. In addition, it was established that they were related to responder index and organ involvement and that TARC stimulated plasmablasts in vitro [76]. Serum plasmablast concentration is a more sensitive marker for the diagnosis of IgG4-RD compared to the serum IgG4 level, however, due to the difficulty of application, it has limited clinical use.

## 4. Treatment

In IgG4-RD, there is a risk of progression from inflammatory and proliferative stage which is responsive to treatments, to fibrotic stage responding weakly to treatments causing severe organ damage. Early diagnosis and treatment are important owing to irreversible organ damage. The aim of treatment is to minimize adverse effects with glucocorticoids and other agents, to produce remission of disease and to preserve organ function. After diagnosis is made, pretreatment evaluation should be made for assessing severity and extension. Routine laboratory tests, whole blood count, kidney and liver function tests, biochemical parameters including amylase and lipase, levels of IgG subtypes, IgE concentration, serum C3 and C4 concentrations, urinalysis (asymptomatic proteinuria may be an indicator of TIN), chest, abdominal and pelvic CT or MRI and PET (for determining the extension of disease) may be ordered. In patients with asymptomatic lymphadenopathy, slight salivary gland enlargement, and incidentally detected lung nodules, watchful waiting policy is recommended. These patients should be evaluated every six months. Patients with symptomatic active IgG4-RD require treatment. Disease activity may be determined by laboratory and imaging methods in addition to symptoms, for example in patients with lacrimal gland swelling, orbital pseudotumor or proptosis. Urgent treatment may be warranted in some patients with pancreatobiliary or renal disease. In addition, patients with aortitis (due to risk of aneurysmal complications such as rupture), RPF (dueto renal failure secondary to ureteral obstruction) pachymeningitis (due to neurological deficit risk), AIP (due to exocrine and endocrine failure) and pericarditis (due to risk of cardiac tamponade) should be urgently treated. In asymptomatic patients with radiological and laboratory findings in vital organs, (e.g., asymptomatic IgG4-RD aortitis/periaortitis, IgG4-RD retroperitoneal fibrosis) treatment should be initiated, since irreversible sequelae may develop [77]. Glucocorticoids are first line agents for remission-induction in treatment naive patients unless they are contraindicated. Initially, prednisone monotherapy is recommended at the dose of 0.6 mg/kg/day (typically 30–40 mg /day). Almost all patients respond to 40 mg daily prednisone within 2–4 weeks. Many patients respond even earlier. After clinical response is obtained at the involved organ, prednisone dose may be gradually tapered during 3–6 months until it is completely discontinued. Rheumatologists from Asia prefer to maintain low dose glucocorticoids (2.5–5mg/day) for three years [78]. Symptomatic improvement upon response to glucocorticoids manifests with decrease in the size of mass or organ enlargement, improvement in organ functions and reduction in serum IgG4 levels. It has been observed that after glucocorticoid induction treatment, CD4 +T cells decline [79]. **** Recurrence was observed in 46% of IgG4-RD patients during and after the tapering of the steroid dose [80]. In selected high-risk patients, i.e. those who have high serum IgG4, IGE and eosinophilia at the onset, those with multiple organ involvement and previous history of relapse, remission maintenance treatment should be contemplated [81]. In a recent retrospective study performed with 277 IgG4-RD patients, recurrence was observed more frequently in patients diagnosed at early ages, those who had allergy history and whose treatment was instituted long time after the diagnosis. Cumulative relapse rates were found to be12.86%, 27.84%, and 36.1%, respectively at 12, 24, and 36 months. As to organs affected by recurrence, (125 organs, 101 patients) recurrence was in de novo organ in 40 patients and in the same organ in 85 patients. The most common de novo recurrence occurred in parathyroid gland. Regarding recurrence in the same organ, the organs where recurrence was most common were lacrimal gland, pancreas and thyroid glands [82]. Following a successful induction treatment, especially some patients with high risk benefited from maintenance treatment [83]. According to international consensus guide, a steroid sparing agent may be administered during maintenance treatment [81]. AZA, MMF, methotrexate, tacrolimus and cyclophosphamide are used as steroid sparing agents [84]. However, there is no agreement on duration of treatment. As active IgG4-RD cases with marked lymphoplasmacytic infiltrationare more likely to respond to pharmacological treatment, in this case the experts recommend immunosuppression. Conversely, in long term stage fibrotic lesions, surgical-debulking should be considered, as they respond poorly to immunomodulatory agents [81]. Spontaneous or at least temporary remissions have been reported [85]. Nevertheless, metachronous nature of the disease suggests that although it may appear to improve transiently in an organ, it may emerge again in a different region month or years later [86]. According to the data obtained in retrospective studies, RTX treatment was effective in cases in which conventional steroid sparing agents were not successful [87]. In patients who are on a steroid regimen when RTX is instituted, the dose of steroid may be reduced following RTX administration [88]. RTX is suitable for maintenance treatment, but the optimal frequency and duration of treatment still remains unclear. Bortezomib (a protease inhibitor) has been used in addition to steroids in recurrent IgG4-RLD [89]. Other biologics, including infliximab, abatacept and tocilizumab, have been reported to be effective in this condition but data are limited to single case reports [90,91]. As described before, IgG4-RD is a fibroinflammatory condition in which delays in treatment can lead to significant fibrosis and damage.

### 4.1. Prognosis-IgG4-related malignancies

The outlook of IgG4-RD depends on the organs involved and the severity of fibrosis. It is known that both hematological and solid organ malignancy risk increases in chronic inflammation, as can be seen in stomach cancer caused by H. Pylori and hepatocellular cancer caused by hepatic viruses. Drawing upon this relation, it may be suggested that there may be a close correlation between IgG4-RD and cancer. In IgG4-RD, the most common hematological and solid organ malignancies are diffuse large cell lymphoma and pancreatic cancer respectively. Considering that IgG4-RD is preponderant in subjects population, who are more immunocompromised, it can be considered as a risk factor for both disease and susceptibility to malignancy. Malignancy may occur before, during or after IgG4-RD. In the study of Asano et al. 158 patients diagnosed with IgG4-RD were followed for a mean of 5.95 ± 4.48 years and malignancy was found in 34 of these patients [92], demonstrating that cumulative risk of cancer was higher than that of general Japanese population, with risk being the highest within the first year. In the same study, malignancy was simultaneously diagnosed with IgG4-RD in 12 patients [93]. High serum total IgG, IgG4, SIL-2R (soluble interleukin 2 receptor) and CIC (circulating immune complex) levels pointed to increased risk of malignancy. In the aforementioned study, 8 patients with malignancy achieved cure with chemotherapy and radiotherapy while remission was obtained in IgG4-RD as well, and they did not recur at follow up evaluations [92]. Therefore, it was thought that IgG4-RD may be a paraneoplastic condition. Yamamoto et al. reported that among 105 patients with IgG4-RD the risk of malignancy was increased in comparison to the general population [93]. Shikawo et al. found the same result in 108 patients with AIP [94]. However, Hart et al. did not find any significant increase in the risk of malignancy in 116 patients with type 1 AIP, as compared to 344 control patients [95]. Likewise, in the study of Hirano et al. on 113 patients with IgG4-RD, malignancy risk was not increased [96]. They attributed this result to the exclusion of patients who had coexistent malignancies at the time of diagnosis. Given the results of aforementioned studies, the relation between malignancies and IgG4-RD is controversial.

## 5. Conclusion

The aim of the present review was to present a general overview on clinical presentations, physiopathology and treatment of IgG4-RD. As IgG4-RD is a relatively new entity the etiology, prevalence and epidemiologic knowledge is quite limited. Although the clinical picture of the disease varies according to the involved organs, compressive symptoms are common due to the tumefactive nature of the disease. IgG4-RD is diagnosed based on the combination of clinical, radiological and histopathological findings. Typical histopathological features include storiform fibrosis, dense lymphoplasmacytic infiltrates and obliterative phlebitis. Serum IgG4 level is neither specific nor sensitive marker for IgG4-RD, but high values have been found to correlate with the severity and recurrence of disease. Glucocorticoids lead to rapid clinical response in most IgG4 **-** RD patients regardless of the clinical picture and organ involvement. For patients requiring treatment, prednisone is usually recommended at an initial dose of 0.6 mg/kg/day (typically 30–40 mg/day). In maintenance treatment, steroid-sparing agents are used both to reduce cumulative risks of steroids and to decrease risk of recurrence. Although association is not clear, malignancies are frequently reported in IgG4-RD patients. Therefore, it is prudent to monitor patients for the symptoms of malignant diseases.
